# Determination and health risk assessment of carbamate pesticide residues in date palm fruits (*Phoenix dactylifera*) using QuEChERS method and UHPLC-MS/MS

**DOI:** 10.1038/s41598-024-63704-6

**Published:** 2024-06-06

**Authors:** Rana Morsi, Kilani Ghoudi, Mohammed A. Meetani

**Affiliations:** 1 Chemistry department, College of Science, United Arab Emirates University, P. O. Box 15551, Al-Ain, United Arab Emirates; 2 Department of Statistics, College of Business and Economics, United Arab Emirates University, P. O. Box 15551, Al-Ain, United Arab Emirates

**Keywords:** Analytical chemistry, Environmental chemistry, Chemical safety

## Abstract

This study aimed to investigate carbamate pesticide residues in different varieties of date palm fruits in the UAE, utilizing UHPLC-MS/MS. For sample preparation and clean-up, the efficiency and performance of different QuEChERS dispersive solid-phase extraction kits were compared. Precision and recovery were assessed at 10 μg kg^−1^ for the three kits, revealing that Kit 2 demonstrated the best performance. The selected QuEChERS method was validated to detect 14 carbamate residues in 55 date samples. The method exhibited strong linearity with R^2^ > 0.999 and low LOD (0.01–0.005 μg kg^−1^) and LOQ (0.003–0.04 μg kg^−1^). Excellent accuracy (recovery: 88–106%) and precision (RSD: 1–11%) were observed, with negligible matrix effect (− 4.98–13.26%). All samples contained at least one carbamate residue. While most detected residues were below their MRLs, carbosulfan was found in 21 samples, propoxur in 2 samples, and carbofuran in 1 sample above their MRLs. The hazard index (HI) was calculated for carbosulfan, phenmedipham, carbaryl, propoxur, carbofuran, and methomyl to assess potential health risks for date consumers. All HI values were below the safety limit of 1.0, indicating that the consumption of dates does not pose a non-carcinogenic health risk for adults and children.

## Introduction

Using pesticides in agriculture has significantly benefited society by protecting crops against unwanted pests and boosting crop yields. However, excessive and improper application of these agrochemicals may increase pesticide residues in agricultural products, posing potential health risks to consumers^1^. Such residues can trigger various environmental impacts, including soil pollution, adverse effects on non-targeted species, and their bioaccumulation and biomagnification within the food chain^2^. Even trace amounts of pesticide residues in agricultural products can accumulate in the human body, potentially leading to detrimental health effects^3,4^. These effects range from short-term issues like headaches, nausea, and rashes to severe conditions such as neurotoxicity, cancer, respiratory disorders, disruptions in the endocrine system, and genetic disorders^2,5–7^.

Carbamate insecticides have gained significant attention in recent years due to their broad biological activity, high effectiveness, relatively low persistence, and comparatively lower toxicity than other pesticide types^6,8–10^. Despite these advantages, carbamates can pose a threat to the nervous system. Their action involves inhibiting the acetylcholinesterase enzyme, leading to an accumulation of acetylcholine at nerve endings. This accumulation can result in neurotoxicity, causing neurological impairments^11,12^. Moreover, some carbamates are suspected to be carcinogenic and mutagenic^12,13^. They have the potential to induce mutations in genes responsible for immune system regulation, impacting immune tolerance and leading to immunotoxicity. Furthermore, they are linked to immune-related diseases such as autoimmune diseases, hypersensitivity reactions, and cancer^11^. The identification of carbamate residues in food has prompted widespread public concern due to their extensive use in crops.

Food safety has emerged as a top global priority. International organizations and governments have implemented rigorous monitoring programs and regulatory frameworks to ensure safe food production. Their primary objective is to regulate pesticide application in food commodities, aiming to reduce pesticide residues' health risks and environmental impacts. Regional or international standards, known as Maximum Residue Limits (MRLs), have been established by the Codex Alimentarius (CA) and the European Union (EU) for this purpose^1,14,15^. MRLs serve as critical benchmarks, determining the maximum acceptable levels of pesticide residues in various food items. These limits are meticulously set to safeguard consumer health and safety^2^.

Date palm fruits (*Phoenix dactylifera*) are widely consumed across various global regions and remain a crucial dietary component in many Arab countries^16^. Dates are highly valued as an excellent energy source, containing many essential minerals and vitamins crucial for human health^3,17^. Moreover, their high levels of antioxidants and antimutagenic contents make them a valuable resource for manufacturing various products, including medicinal items and beauty products^18^. Additionally, they have been explored for their medicinal potential in treating cancer and several infectious diseases^19^. Investigations into date fruits have highlighted their potential in fruit sugar production, a healthy and nutritious alternative to commercially refined sugar^20^. Furthermore, extensive research has focused on various beneficial applications using date seeds extracted from the fruit. These applications include the production of activated carbon for water treatment^21–23^, cellulase production^24^, and biohydrogen synthesis^25^.

Dates are commonly cultivated in arid and semi-arid regions, such as the Middle East and North Africa, where necessary conditions for successful growth, such as hot climates, dry weather, abundant sunlight, and well-drained soil, are prevalent. According to the Food and Agriculture Organization (FAO), the United Arab Emirates (UAE) ranks among the top 10 countries for date production, yielding approximately 397,328 tons/year^26^. Over the past two decades, date palm cultivation has significantly expanded in the UAE, encompassing more than 250 types of date crops across various regions^17^. This substantial increase in date fruit production is paralleled by a high consumption rate within the UAE, where individuals consume an average of 114.3 g (equivalent to approximately 10 date fruits) per capita daily^16^. Like many other fruits, dates are susceptible to contamination by pesticide residues. Given the considerable consumption of dates, there is an essential need to examine the presence of pesticide residues within this fruit to uphold food safety standards and protect public health. Consuming dates contaminated with pesticides can harm human health, emphasizing the criticality of implementing rigorous monitoring and control measures to mitigate potential risks associated with their consumption.

The analysis of pesticide residues in food samples typically involves utilizing liquid chromatography (LC) and gas chromatography (GC) coupled with various detectors, such as mass spectrometry (MS), tandem mass spectrometry (MS/MS), and electron capture detector (ECD). LC–MS/MS^3,4,27^, GC–MS^14^, and GC–MS/MS^28^ have been employed to determine pesticide residues in dates. However, analyzing dates poses challenges due to numerous interfering compounds that significantly impact the analysis and the overall performance of the analytical method. Therefore, a critical step in this process involves sample preparation, including extraction and clean-up. The quick, easy, cheap, effective, rugged, and safe (QuEChERS) technique stands out due to its advantages over traditional techniques. This method offers simplicity, high recovery for a broad spectrum of pesticides, minimal waste and solvent usage, and cost-effectiveness, and it delivers reliable, precise, accurate results^4,29^.

A limited amount of research has been dedicated to determining pesticide residues in dates on a global scale^3,4,14,27,28^. To our knowledge, no published research has been conducted on detecting pesticide residues in dates cultivated in the UAE. Therefore, this study aims to investigate the residual levels of 14 different carbamate pesticides in various types of dates in the UAE and compare them to the CA- and EU-MRLs. We used a simple QuEChERS method for the extraction and clean-up of carbamate residues from samples, followed by their quantification using UHPLC-MS/MS. Furthermore, we conducted a probabilistic assessment using Monte-Carlo simulation to evaluate the health risks associated with some of the detected carbamates for adults and children.

## Materials and methods

### Chemicals and reagents

High purity carbamate pesticide standards were obtained from Dr. Ehrenstorfer (Augsburg, Germany). The internal standard (IS), pirimicarb d6, was purchased from Sigma-Aldrich (St. Louis, MO, USA). LC–MS grade acetonitrile and methanol were procured from Honeywell (Seelze, Germany). Ultrapure water was produced using the Milli-Q Plus system (MilliporeSigma, USA). Ammonium formate, sodium chloride (NaCl), anhydrous magnesium sulfate (MgSO_4_), and glacial acetic acid were supplied by Sigma-Aldrich (St. Louis, MO, USA). All QuEChERS dispersive solid-phase extraction (d-SPE) salts were provided by Agilent Technologies Inc. (Wilmington, DE, USA). Millex PTFE syringe filters, used for sample filtration, were purchased from Merck Millipore (Carrigtwohill, Ireland).

### Preparation of standards and calibration curves

Individual stock solutions (200 mg kg^−1^) for each carbamate pesticide were prepared using appropriate organic solvents based on their solubility. Working standard solutions for optimization and mixed solutions utilized for method development, calibration, and method validation were prepared by diluting the stock solutions in methanol. The stock and working solutions were stored in the dark at − 18 °C.

Calibration curves were prepared in methanol and date matrix to assess the matrix effect. These curves were obtained using 12 data points covering concentrations of 0.001, 0.005, 0.01, 0.05, 0.1, 0.5, 1.0, 5.0, 10, 50, 100, 500 μg kg^−1^. A fixed concentration of pirimicarb-d6 IS (100 μg kg^−1^) was added to all samples. The calibration curves were constructed by correlating the relative responses of each carbamate pesticide, represented by the ratio of the compound’s peak area to the IS, across a range of known concentrations (0.001–500 μg kg^−1^) of the compounds.

### Sample collection and preparation

A total of 55 fresh date samples were collected from various markets and local farms across the UAE, encompassing different varieties cultivated and consumed in the region. Samples from different farms were placed in sterile polypropylene bags and appropriately sealed to prevent contamination during transport to the laboratory. Upon arrival, samples were refrigerated at 4 °C until analysis to maintain freshness. The collection of date samples complied with the European SANTE/11,312/2021 guidelines^30^. The study was conducted following relevant guidelines and regulations. Permissions and approvals were obtained from farm owners prior to sample collection.

Caps and seeds were removed for sample preparation, and 50 g (sufficient for triplicate extractions and analyses) of each date sample was transferred to a food processor. Then, 75 mL (1.5 times the sample’s weight) of cold deionized water was added to the dates, and the mixture was homogenized in the food processor at high speed for 3 min, resulting in a homogenized date paste. All handling, storage, preparation, and processing procedures adhered to the European SANTE/11,312/2021 guidelines^30^.

### Extraction and clean-up

10 g of homogenized date paste were weighed and transferred to 50 mL polypropylene centrifuge tubes. Subsequently, 100 μL of pirimicarb d6 (1.0 mg kg^−1^) was pipetted into the date paste. Following this, 10 mL of acetonitrile with 1% acetic acid (v/v) was added to the samples, and mixtures were shaken for 3 min. The tubes were then incubated in a dark place at 4 °C for 60 min. After incubation, 4 g of anhydrous MgSO_4_ and 1 g of NaCl were added to the samples. The samples were vortexed for 3 min and centrifuged at 4500 rpm for 10 min. Afterward, the supernatant was transferred to 15 mL QuEChERS d-SPE tubes. These tubes were vortexed for 2 min and centrifuged at 4500 rpm for 10 min. After the centrifugation, the extract was transferred to a glass tube, evaporated, and reconstituted with 1 mL of mobile phase B (mixture of methanol and acetonitrile 2:1). Finally, the extract was filtered through a 0.45 μm PTFE syringe filter and analyzed using UHPLC-MS/MS.

An assessment was conducted to evaluate the efficiency and performance of three distinct QuEChERS d-SPE kits in extracting carbamate pesticide residues from the date matrix. Each kit comprised different combinations of sorbents and salts. Kit 1 contained 150 mg primary secondary amine (PSA), 150 mg octadecylsilane chemically bonded silica, endcapped (C18EC), and 900 mg MgSO_4_. Kit 2 consisted of 400 mg PSA, 400 mg graphitized carbon black (GCB), 400 mg C18EC, and 1200 mg MgSO_4_. Finally, Kit 3 included 400 mg PSA, 400 mg C18EC, and 1200 mg MgSO_4_. The efficiency of the three different QuEChERS d-SPE kits was determined by extracting the same date sample and comparing the number of detected carbamate residues. Additionally, the performance of the QuEChERS d-SPE kits was assessed by spiking blank date samples with carbamate standards at a concentration of 10 μg kg^−1^ to evaluate recovery and precision.

### UHPLC-MS/MS instrumentation and conditions

The analytical determination of carbamate residues in dates was performed using a Shimadzu UHPLC instrument (Nexera-i LC-2040C 3D, Kyoto, Japan) coupled with an 8030 Shimadzu triple quadrupole LCMS (Kyoto, Japan). LC separation was carried out on an ACQUITY UPLC BEH C18 column (2.1 mm × 150 mm × 1.7 μm) provided by Waters (Milford, USA), with the column temperature maintained at 45 °C. The mobile phase comprised (A) 10 mM ammonium formate in water (pH = 3) and (B) a mixture of methanol and acetonitrile (2:1). The total runtime was 23 min, utilizing a gradient elution program summarized in Table 1. The flow rate was set at 0.15 mL min^−1^, and the injection volume was 15 μL. Tandem MS detection was performed using an electrospray ionization (ESI) source in positive mode, optimizing the multiple reaction monitoring (MRM) mode to enhance specificity and sensitivity. Operational settings included a nebulizer gas flow of 3.0 L/min, dry gas flow of 15 L/min, a DL temperature of 250 °C, a heat block temperature of 400 °C, a CID gas pressure at 230 kPa, and interface voltage at 4.5 kV. Detailed MS/MS parameters for each analyte are available in Supplementary Table S1 online.

**Table 1:** Gradient elution program.

Time (min)	Mobile phase A: 10 mM ammonium formate in water (%)	Mobile phase B: a mixture of methanol and acetonitrile (2:1) (%)
0.00–4.00	85	15
4.00–6.00	15	85
6.00–14.00	15	85
14.00–15.00	5	95
15.00–17.00	5	95
17.00–20.00	85	15
20.00–23.00	85	15

### Matrix effect

To assess the matrix effect, calibration curves for 14 carbamate pesticides were constructed at 12 concentrations ranging from 0.001 to 500 μg kg^−1^ in pure solvent (methanol) and blank date matrices. The relative responses of each carbamate to the IS, as mentioned in Section “Preparation of standards and calibration curves”, were used to establish these curves. The matrix effect was quantified by comparing the slopes of calibration curves prepared in solvent and date matrices using the following formula:

$$\text{Matrix effect }\left({\%}\right)=\frac{slope\left(matrix-matched \,curve\right)-slope (solvent-based \,curve)}{slope (solvent-based \,curve)} \times 100\%$$

Matrix effect values can be negative, indicating ion suppression, or positive, indicating ion enhancement. If matrix effect values range from − 20 to 20%, the effect is considered negligible and is referred to as soft matrix effect. In this case, a solvent-matched calibration curve can be used, which is easier, simpler, and less time-consuming than the matrix-matched calibration curve. Conversely, matrix effect values outside this range (below − 20 or above 20) suggest a medium or strong matrix effect. A matrix-matched calibration curve should be used in such cases as matrix effects are considered significant^31^.

### Validation study

The analytical method was validated in terms of linearity, limits of detection (LOD) and quantitation (LOQ), recovery, repeatability, and intermediate precision following the European SANTE/11,312/2021 guidelines^30^.

Linearity was assessed using solvent-matched calibration curves over a concentration range from 0.001 to 500 μg kg^−1^. Excellent linearity was demonstrated by a high coefficient of determination (R^2^).

The LOD and LOQ were used to evaluate the analytical method’s sensitivity and accuracy. LOD and LOQ are the minimum concentrations at which an analyte can be reliably detected and quantified, respectively. In our study, we determined the LOD and LOQ based on the standard deviation of the blank measurements. We performed 20 measurements of blank samples to establish the mean blank signal $$({\overline{S}}_{bl})$$ and its standard deviation ($${\sigma }_{bl})$$. The minimum distinguishable analytical signal ($${S}_{m})$$ was then calculated using the formula:

$${S}_{m}={\overline{S} }_{bl}+k{\sigma }_{bl}$$

where $k$ is set to 3 for LOD and 10 for LOQ. This corresponds to a signal-to-noise ratio (S/N) greater than 3 for LOD and greater than 10 for LOQ. To convert ${S}_{m}$ to the corresponding minimum concentration (${c}_{m}$), which is defined as the detection or quantitation limits, the following formula was used:

$${c}_{m}=\frac{{S}_{m}- {\overline{S} }_{bl}}{m}$$

where $m$ is the slope of calibration curves. Simplifying this, since ${S}_{m}-{\overline{S} }_{bl}=k{\sigma }_{bl}$, we drive that:

$${c}_{m}=k\frac{ {\overline{S} }_{bl}}{m}$$

This calculation allows ${c}_{m}$ to represent the LOD when $k$= 3 and the LOQ when $k$ = 10. The approach for calculating LOD and LOQ follows the methodology described in Skoog et al. (32, p.20)^32^.

For the recovery assessment, blank date samples were spiked with a mixture of carbamate standards at concentrations of 0.5 μg kg^−1^ and 10 μg kg^−1^ in triplicates. Per the SANTE/11,312/2021 guidelines^30^, acceptable recoveries should fall within the 60–140% range. The following formula was used to calculate recoveries:

$$\text{Recovery }\left({\%}\right)=\frac{Amount\, of\, analyte\, measured}{Amount\, of\, analyte\, spiked } \times 100$$

Precision was evaluated through intraday (repeatability) and interday (intermediate precision) analyses. Six replicates were analyzed within a single day for repeatability, while 12 replicates (6 analyses per day) were conducted over two consecutive days for intermediate precision at concentration levels of 0.5 μg kg^−1^ and 10 μg kg^–1^. Precision is evaluated via the relative standard deviation (RSD), calculated using the formula:

$$\text{RSD }\left(\text{\%}\right)=\frac{Standard deviation}{Mean}\times 100$$

The RSD should be ≤ 20% to comply with the European SANTE/11,312/2021 guidelines^30^.

### Uncertainty measurement

Measurement uncertainty (MU) refers to the dispersion of results associated with every measurement, providing a range of values within which the actual value is expected to be found. In the analysis of pesticide residues, MU is a crucial parameter^33^. According to the European Commission SANTE/11,312/2021 guidelines, MU values should not exceed 50%^30^. In this study, we estimated MU based on intra-laboratory validation data. MU, referred to as $\stackrel{`}{U}$, was calculated using the following formula:

$$\mathop U\limits^{\prime } = k\mathop u\limits^{\prime }$$

where $k$ is the coverage factor, assumed to be 2, and *ú* is the relative standard uncertainty. The standard uncertainty was measured using trueness (bias) and precision and was estimated for each carbamate pesticide using the following formula:

$$\mathop u\limits^{\prime } = \sqrt {\mathop u\limits^{\prime } \left( {bias} \right)^{2} + \mathop u\limits^{\prime } \left( {precision} \right)^{2} }$$

where $\mathop u\limits^{\prime } \left( {bias} \right)$ is the uncertainty due to bias, estimated from recovery experiments, and $\mathop u\limits^{\prime } \left( {precision} \right)$ is the uncertainty for the precision obtained from precision experiments. The bias, which refers to the differences between measured and true values, was calculated as follows:

$$\mathop u\limits^{\prime } \left( {bias} \right) = \sqrt {mean^{2}_{bias} + SD.P^{2}_{bias} }$$

where ${{mean}^{2}}_{bias}$ is the mean of the relative bias and $SD.{{P}^{2}}_{bias}$ is the population standard deviation of the relative bias. The value of *ú*(*precision*) can be estimated from the %RSD of the reproducibility data for each pesticide and was calculated as follows:

$$\mathop u\limits^{\prime } \left( {precision} \right) = \% RSD$$

### Health risk assessment

A probabilistic approach utilizing Monte-Carlo simulation was employed to assess human health risks^34^. Monte-Carlo was utilized to generate distributions of carbamate pesticide residues. The distribution of each pesticide was selected to reflect the data obtained from our samples. We chose the lognormal distribution and picked the mean and standard deviation equal to those obtained from our samples. In the context of our study, we conducted 100,000 replicates. Hazard quotients (HQ) were computed for each simulation replicate, and the 95th percentile of HQ distribution was used for risk assessment. HQ calculations were performed for both adults and children using the following approach.

First, the critical daily intake (CDI) was calculated as follows:

$$CDI=\frac{C*IR}{BW}$$

where $C$ is the carbamate pesticide residue concentration in date samples (measured in mg kg^-1^), $IR$ stands for the ingestion rate of dates by consumers, and $BW$ represents the body weight taken as 70 kg for adults and 15 kg for children. The $IR$ of dates in the UAE was reported by Ismail et al. to be 114.3g/day for adults. While $IR$ for children was not reported, Ismail et al. noted a decline in date consumption among younger population groups. Using our estimation, we chose an $IR$ of 3 dates per day, equivalent to 35 g per day, for children.

Next, the non-cancerous risk HQ was determined by:

$$HQ=\frac{CDI}{RfDo}$$

where $RfDo$ represents the pesticide chronic oral reference dose obtained from the US-EPA (https://iris.epa.gov/AtoZ/?list_type=alpha). Finally, the hazard index (HI) was obtained as the sum of the HQ values for the pesticides studied. As noted by Boobis, pesticides present a non-carcinogenic risk if HQ or HI < 1, while HQ or HI > 1 indicates potential non-carcinogenic risk due to pesticide residues^35^. In our study, we used $RfDo$ values of 0.01, 0.25, 0.1, 0.005, 0.005, and 0.25 mg/kg/day for carbosulfan, phenmedipham, carbaryl, propoxur, carbofuran, and methomyl, respectively. Risk assessments were solely conducted for these specified pesticides, as $RfDo$ values for the remaining pesticides are unavailable.

## Results and discussion

### QuEChERS method selection

**Figure 1:**
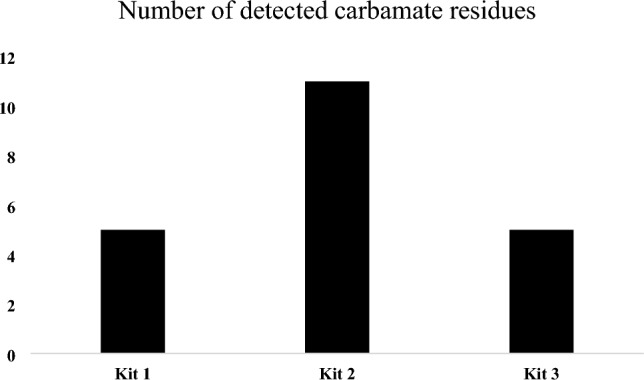
Number of detected carbamate residues using three different QuEChERS d-SPE kits.

A comparative analysis of three distinct QuEChERS d-SPE kits was conducted to determine the optimal kit for extracting carbamate pesticides from dates. The efficiency was evaluated by extracting carbamate residues from the same date sample using each of the three kits and then comparing the number of detected residues, as shown in Fig. 1. Kits 1 and 3 successfully extracted five carbamate residues. Notably, these kits extracted the same carbamate types: carbosulfan, propoxur, aminocarb, pirimicarb, and fenoxycarb. On the other hand, Kit 2 extracted 11 carbamate residues. In addition to the 5 carbamates mentioned above, it extracted phenmedipham, carbaryl, bendiocarb, carbofuran, desmedipham, and methiocarb, indicating superior extraction efficiency.

The performance of each QuEChERS d-SPE kit was evaluated by spiking blank date samples with a mixture of the 14 carbamate standards at 10 μg kg^−1^ and comparing the obtained recovery and precision (expressed as RSD%). Table 2 summarizes the recoveries and RSD values obtained from the three kits. Kit 2 exhibited the most consistent and favorable performance. It demonstrated recoveries ranging from 89 to 103%, indicating reliable and efficient extraction capability. Moreover, its RSD values consistently remained low, ranging from 1 to 8%, demonstrating high precision in the extraction procedure. In comparison, Kits 1 and 3 displayed more varied performance. Kit 1 showed acceptable recoveries for some compounds; however, it exhibited recoveries below the acceptable range for phenmedipham (43%), carbaryl (59%), bendiocarb (53%), desmedipham (41%), and methiocarb (29%). Additionally, there was a fluctuation in RSD values, with some compounds displaying RSD > 20%, such as propamocarb, bendiocarb, and methiocarb. Similarly, Kit 3 demonstrated recovery values outside the acceptable range for some compounds, such as bendiocarb (45%), methomyl (54%), desmedipham (59%), and methiocarb (16%). It also exhibited high variation across RSD values, with carbaryl, methomyl, and methiocarb showing RSD > 20%. Based on the results, Kit 2 exhibited superior efficiency in the extraction and clean-up processes of carbamate pesticides from the date matrix. It showed a more consistent and robust performance across all evaluated carbamates, meeting the recovery and precision criteria of the European SANTE/11,312/2021 guidelines, compared to Kits 1 and 3.

**Table 2:** Performance parameters of three different QuEChERS d-SPE kits at 10 μg kg^-1^.

Compound	Kit 1		Kit 2		Kit 3	
	% Recovery	% RSD	% Recovery	% RSD	% Recovery	% RSD
Carbosulfan	101	2	99	3	97	5
Phenmedipham	43	11	94	3	65	3
Carbaryl	59	5	100	2	72	27
Propoxur	97	2	99	1	100	1
Propamocarb	72	21	97	6	97	6
Aminocarb	101	1	99	1	101	3
Ethiofencarb	93	6	100	4	76	11
Pirimicarb	100	1	103	2	100	2
Bendiocarb	53	33	94	8	45	5
Fenoxycarb	98	1	102	1	100	1
Carbofuran	77	2	99	1	79	4
Methomyl	90	2	98	3	54	34
Desmedipham	41	6	99	3	59	6
Methiocarb	29	24	89	8	16	39

The improved performance of Kit 2 can be credited to its unique blend of clean-up sorbents, which are optimized for the complex matrix of date palm fruits. Kit 2 contains 400 mg each of PSA, GCB, C18EC, and 1200 mg of MgSO_4_. The presence of GCB is particularly noteworthy, as it is absent in Kits 1 and 3. GCB is known for its high surface area and strong adsorptive capacity, making it highly effective in removing pigments and sterols from complex matrices^36^. Date palm fruits are rich in carotenoids and sterols^37^, which can interfere with carbamate detection. The ability of GCB to eliminate such interferences has significantly contributed to the efficacy of Kit 2. Additionally, the synergistic combination of GCB with PSA and C18EC may be ideal for handling the complexity of the date matrix. While PSA targets polar organic acids, sugars, and fatty acids, and C18EC attracts non-polar compounds^36^, GCB effectively bridges the gap by targeting moderately polar to non-polar interferences, thus offering a broader spectrum of clean-up efficiency. As mentioned, Kit 2 did not only detect a greater number of carbamate residues (11 compared to 5 in Kits 1 and 3, as illustrated in Fig. 1) but also maintained recoveries within the acceptable range of 60–140% and RSDs below 20% (as detailed in Table 2). These findings underline the robustness of Kit 2 in addressing the specific challenges posed by the date matrix. During the experiment, we observed the impact of GCB on removing pigments present in dates. Before the clean-up steps, the extracts had a yellow/brown color. After the clean-up step, only the extract from Kit 2 was clear, whereas the extracts remained yellow/brown when using Kits 1 and 3. Including GCB in Kit 2, combined with higher amounts of other sorbents and MgSO_4_, provides a more effective clean-up action, which is more suited to the characteristics of date matrices. Therefore, due to its consistently higher recovery rates and precision, Kit 2 was selected for the extraction and clean-up processes of the carbamate residues in dates using UHPLC-MS/MS.

### Matrix effect

One of the significant challenges encountered when utilizing UHPLC-MS for analyzing complex natural matrices, such as dates, is the matrix effect. During the ionization of target analytes, interferences may arise due to co-eluting compounds present in the matrix. These interferences can lead to alterations in the signal, appearing either as a decrease (ion suppression) or an increase (ion enhancement), ultimately impacting the analytical method’s performance^4,38,39^. Various strategies have been proposed to address this issue, including using isotopically labeled or deuterated IS, employing standard addition or matrix-matched calibration curves, and implementing appropriate sample preparation and clean-up methods^39^. Additional approaches to minimize the matrix effect involve reducing the injection volume, diluting the sample, decreasing the flow rate, and extending the gradient program^39,40^.

The matrix effects were determined for all 14 carbamate pesticides (Table 3). Among the studied carbamates, carbosulfan, carbaryl, propoxur, and bendiocarb resulted in soft signal suppression with matrix effect values of − 4.46%, − 4.98%, − 3.29%, and − 0.96%, respectively. On the other hand, the remaining 10 carbamates resulted in soft signal enhancement with matrix effect values ranging from 0.28 to 13.26%. All obtained matrix effects are <  ± 20, demonstrating negligible effects according to the European SANTE/12,830/2020 guidelines^41^. Hence, solvent-matched calibration curves can be used. The observed negligible matrix effect can be attributed to appropriate sample preparation and clean-up methodology and the use of IS, which offset the dependence of results obtained on the matrix.

**Table 3:** Validation parameters including coefficient of determination, LOD, LOQ, and matrix effect.

Carbamate	R^2^	Linear dynamic range (μg kg^−1^)	LOD (μg kg^−1^)	LOQ (μg kg^−1^)	Matrix effect (%)
Carbosulfan	0.9999	0.01–500	0.0107	0.0323	− 4.46
Phenmedipham	0.9998	0.001–100	0.0015	0.0044	10.92
Carbaryl	0.9997	0.001–100	0.0014	0.0043	− 4.98
Propoxur	0.9998	0.001–500	0.0011	0.0034	− 3.29
Propamocarb	0.9999	0.005–500	0.0049	0.0149	13.26
Aminocarb	0.9999	0.001–500	0.0010	0.0031	1.77
Ethiofencarb	0.9999	0.001–500	0.0011	0.0033	4.40
Pirimicarb	0.9999	0.005–100	0.0053	0.0162	0.71
Bendiocarb	0.9995	0.001–100	0.0014	0.0043	− 0.96
Fenoxycarb	0.9998	0.001–500	0.0011	0.0033	5.95
Carbofuran	0.9998	0.001–500	0.0011	0.0035	1.80
Methomyl	0.9998	0.01–500	0.0131	0.0398	4.82
Desmedipham	0.9998	0.001–500	0.0014	0.0043	0.71
Methiocarb	0.9999	0.001–500	0.0013	0.0041	0.28

### Validation study

The selected QuEChERS method was validated following the European SANTE/11,312/2021 guidelines by evaluating linearity, LOD, LOQ, recovery, repeatability, and intermediate precision.

The linearity of the method was evaluated by constructing calibration curves across a concentration range of 0.001–500 μg kg^−1^ and measuring peak areas relative to the IS. Excellent linearity was observed for all carbamate pesticides, with R^2^ > 0.999 (Table 3). The LOD and LOQ values are presented in Table 3. The LOD (S/N ≥ 3) was approximately 0.01 μg kg^−1^ for carbosulfan and methomyl, 0.005 μg kg^−1^ for propamocarb and pirimicarb, and 0.001 μg kg^-1^ for the remaining compounds, while the LOQ (S/N ≥ 10) ranged from 0.003 to 0.04 μg kg^−1^. LOD and LOQ values were below the MRLs, indicating the method’s sensitivity to quantify trace levels of carbamate residues in dates.

The recovery and precision results are listed in Table 4. Recoveries were determined by adding a mixture of carbamate standards at concentrations of 0.5 μg kg^−1^ and 10 μg kg^−1^, and analyzing three replicates using UHPLC-MSMS at each spiked concentration level. At 0.5 μg kg^−1^, recoveries for all carbamate pesticides ranged from 88 to 106%, while at 10 μg kg^−1^, recoveries ranged from 89 to 103%. The obtained recoveries for low and high concentrations were within the acceptable range, suggesting a consistent and reliable method. Precision was evaluated in terms of repeatability (intraday) and intermediate precision (interday). The RSD of replicates for intraday and interday analyses ranged from 1 to 11%, falling within the acceptable range (RSD ≤ 20) and meeting the SANTE/11,312/2021 guidelines^30^.

**Table 4:** Recovery, repeatability, and intermediate precision at two concentrations.

Carbamate	0.5 μg kg^−1^			10 μg kg^−1^		
	% Recovery	% RSD Intraday	% RSD Interday (n = 2)	% Recovery	% RSD Intraday	% RSD Interday (n = 2)
Carbosulfan	102	1	1	99	1	3
Phenmedipham	93	9	7	94	3	3
Carbaryl	102	3	3	100	2	2
Propoxur	98	8	5	99	1	1
Propamocarb	100	5	3	97	8	6
Aminocarb	99	6	4	99	2	1
Ethiofencarb	101	4	2	100	5	4
Pirimicarb	106	4	4	103	3	2
Bendiocarb	99	3	5	94	10	8
Fenoxycarb	105	6	4	102	1	1
Carbofuran	97	4	3	99	2	1
Methomyl	96	4	3	98	2	3
Desmedipham	99	9	8	99	2	3
Methiocarb	88	11	11	89	8	8

The validation of the selected QuEChERS method demonstrated robustness and reliability across all evaluated merit figures. The results indicate the method’s suitability for accurately and sensitively determining trace levels of carbamate residues in dates.

### Uncertainty measurements

The expanded MU, referred to as $\stackrel{`}{U}$, was determined by multiplying the standard uncertainty (*ú*) by a coverage factor ($k$) of 2, which approximately corresponds to a confidence level of 95%. To follow the European SANTE/11,312/2021 guidelines, MU should not exceed the default value of 50%, a threshold typically used by regulatory authorities when making enforcement decisions. The MU was estimated at two concentration levels: 0.5 μg kg^−1^ (see Supplementary Fig. S1 online) and 10 μg kg^-1^ (see supplementary Fig. S2 online). The MU values of all carbamate pesticides did not exceed the default value of 50% at both concentration levels, indicating that our results are accurate and reliable.

### Analysis of date samples

After validating the method, 55 date samples were analyzed from various markets and farms across the UAE. The levels of 14 different carbamate pesticides were measured using UHPLC-MS/MS in these date samples and compared against their MRLs. Table 5 summarizes the distribution of carbamate residues found in the date samples. The number and concentration of identified carbamate residues varied among the samples. Every sample contained at least one carbamate residue, and each carbamate pesticide was detected in at least one sample (see Supplementary Table S2 online). Most of the detected carbamate residues were found at levels below their respective MRLs. Carbosulfan, propoxur, and carbofuran were the only carbamates detected at levels above their MRLs. Carbosulfan was detected in all 55 samples (100%), with concentrations ranging from 2.54 to 24.77 μg kg^−1^. Its concentration exceeded the MRL (10 μg kg^−1^) in 21 samples (38.2%). The mean concentration of carbosulfan (10.33 ± 0.19 μg kg^−1^) indicated widespread contamination, posing a potential health risk to consumers. Propoxur was detected in 53 samples (96.4%), with concentrations ranging from 0.73 to 6.08 μg kg^−1^, exceeding its MRL (5 μg kg^−1^) in only two samples (3.6%). Carbofuran was identified in 33 samples (60%), with concentrations ranging from 0.13 to 3.27 μg kg^−1^, surpassing its MRL (3 μg kg^−1^) in only one sample (1.8%). The other carbamate pesticides were detected at varying frequencies, but their concentrations were below the MRLs established by the EU and CA. High frequencies of detection were observed for phenmedipham (detected in 46 samples, equivalent to 83.6%), propamocarb (43 samples, 78.2%), aminocarb (52 samples, 94.5%), and ethiofencarb (51 samples, 92.7%). Pirimicarb, bendiocarb, and fenoxycarb were found in 27 (49%), 22 (40%), and 31 (56.4%) samples. Carbaryl, methomyl, desmedipham, and methiocarb were detected in less than 30% of the samples. The results indicate varying concentrations of carbamate residues in the date samples, highlighting the significance of continuous monitoring and strict regulations to ensure consumer safety.

**Table 5:** Distribution of carbamate residues found in date samples.

Pesticide	Contaminated samples No. (%)	> MRL No. (%)	Range (min–max) (μg kg^-1^)	Mean concentration ± STD (μg kg^-1^)	MRL^b^ (μg kg^-1^)
Carbosulfan	55 (100%)	21 (38.2%)	2.54–24.77	10.33 ± 0.19	10
Phenmedipham	46 (83.6%)	NA^a^	0.10–4.23	0.63 ± 0.03	10
Carbaryl	14 (25.5%)	NA	0.04–1.65	0.46 ± 0.02	10
Propoxur	53 (96.4%)	2 (3.6%)	0.73–6.08	1.48 ± 0.06	5
Propamocarb	43 (78.2%)	NA	0.28–1.21	0.51 ± 0.02	10
Aminocarb	52 (94.5%)	NA	0.06–3.28	0.60 ± 0.02	10
Ethiofencarb	51 (92.7%)	NA	0.34–3.53	1.28 ± 0.06	10
Pirimicarb	27 (49%)	NA	0.19–3.93	0.99 ± 0.04	10
Bendiocarb	22 (40%)	NA	0.06–1.85	0.49 ± 0.02	10
Fenoxycarb	31 (56.4%)	NA	0.03–5.93	1.49 ± 0.04	10
Carbofuran	33 (60%)	1 (1.8%)	0.13–3.27	0.85 ± 0.03	3
Methomyl	15 (27.3%)	NA	0.22–1.70	0.86 ± 0.02	10
Desmedipham	3 (5.5%)	NA	0.58–4.43	2.93 0.13	10
Methiocarb	8 (14.5%)	NA	0.03–1.24	0.39 + 0.02	30

^a^NA: Not available above the MRL.

^b^MRL: according to CA and EU^42,43^.

### Health risk assessment

**Figure 2:**
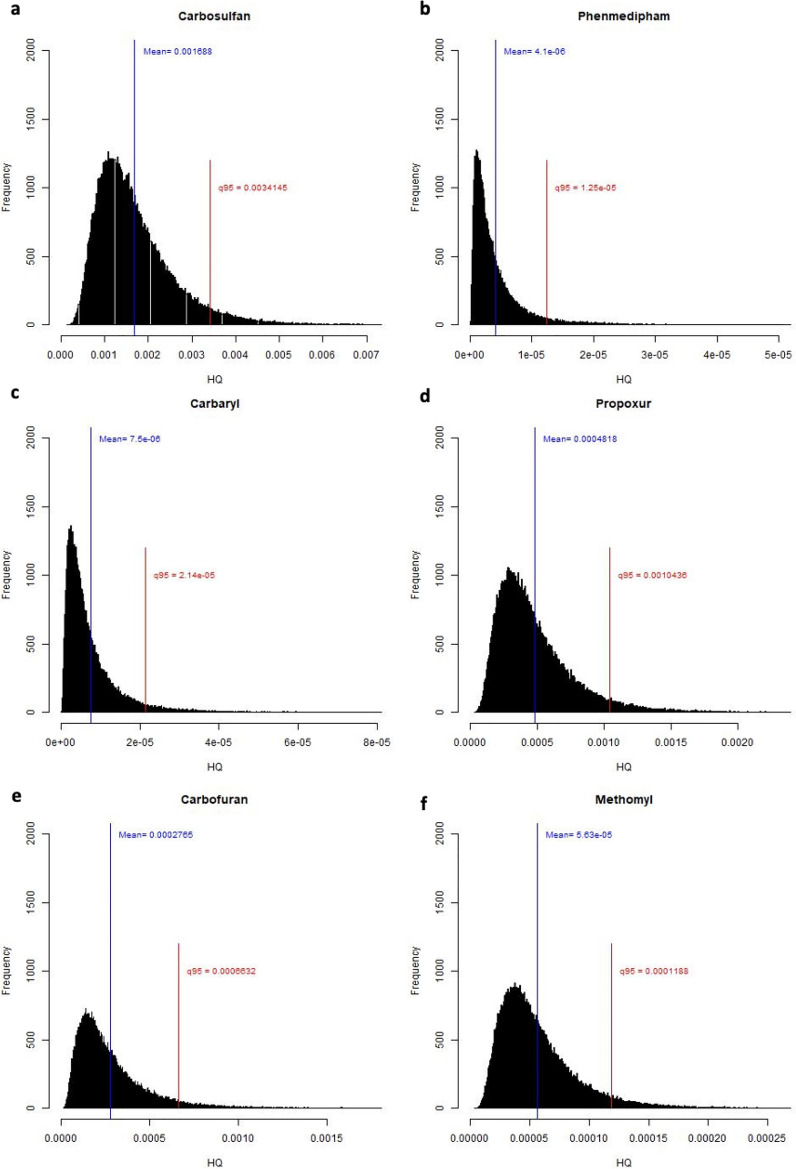
HQ due to residual levels of (**a**) carbosulfan; (**b**) phenmedipham; (**c**) carbaryl; (**d**) propoxur; (**e**) carbofuran; and (**f**) methomyl in date samples for adults.

**Figure 3:**
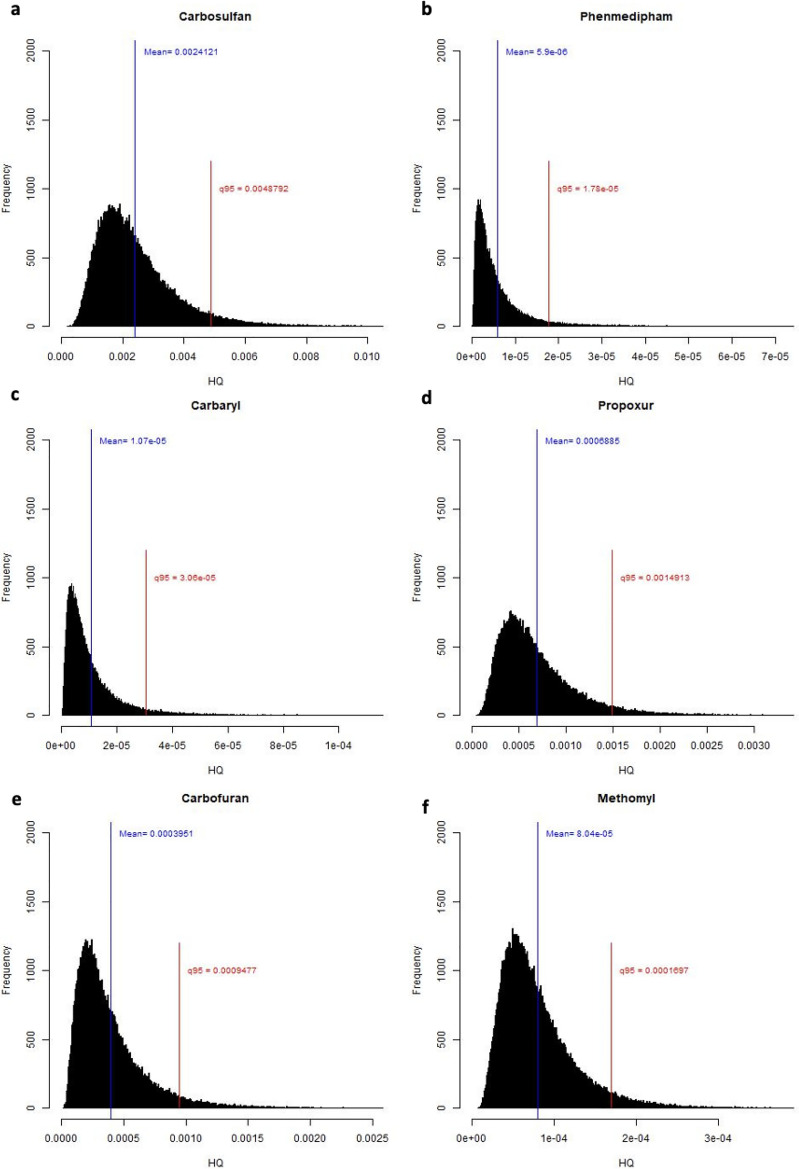
HQ due to residual levels of (**a**) carbosulfan; (**b**) phenmedipham; (**c**) carbaryl; (**d**) propoxur; (**e**) carbofuran; and (**f**) methomyl in date samples for children.

The older generations in the UAE consume dates daily, whereas this tradition is less prevalent among younger Emiratis. Generally, the detected amounts of pesticide residues in dates are below the MRL set by the CA and EU. However, considering the regular consumption of dates by the adult population, it becomes crucial to assess the long-term health risks posed by pesticides found in date samples. Our study analyzed the non-carcinogenic health risks associated with six pesticides: carbosulfan, phenmedipham, carbaryl, propoxur, carbofuran, and methomyl. Figure 2 displays the simulation results for the distributions of HQ for these pesticides among adults. The figure depicts the mean and the 95th percentile for each distribution. The 95th percentiles of the HQ distributions, used here to assess health risk, are reported in Supplementary Table S3 online. Notably, all these percentiles are well below the safety limit of 1.0. To assess the combined risk of these pesticides, we computed the HI as the sum of the 95th quantiles for all considered pesticides. Our findings indicated an HI value of 0.00524, which is less than 1.0. Consequently, the consumption of dates in the UAE does not pose a non-carcinogenic health risk for the adult population. Furthermore, we conducted a risk assessment for children. The results, depicted in Fig. 3, indicate that none of the HQ quantile values, reported in Supplementary Table S4 online, exceeds the safety limit of 1.0. The calculated HI for children was 0.00749, also below the safety limit of 1.0. These findings suggest that the consumption of dates does not pose a non-carcinogenic health risk for children in the UAE.

## Conclusion

This study investigated 14 different carbamate pesticides across various types of date samples using UHPLC-MS/MS. A simple QuEChERS method was used for sample preparation and clean-up. The method was validated and applied to 55 distinct date samples collected from various markets and farms across the UAE. The method showed excellent linearity (R^2^>0.999 for all carbamate pesticides), low limits of detection and quantification (< MRLs), along with acceptable recoveries (88–106%) and precision (RSD ≤ 20%). Matrix effects were evaluated, with values ranging between − 4.98 and 13.26%, indicating a negligible effect. The majority of detected carbamate residues in date samples were below their respective MRLs, except for carbosulfan (>MRL in 21 samples), propoxur (>MRL in 2 samples), and carbofuran (>MRL in 1 sample). Non-carcinogenic health risks associated with some of the detected carbamate pesticides were assessed for adults and children. The obtained HI < 1.0 indicates that date consumers are not at risk.

### Supplementary Information

Supplementary Information.

## Data Availability

The datasets generated during and/or analyzed during the current study are available from the corresponding author on reasonable request.
